# Multi-Informant Universal Mental Health Screening for Preschool-Aged Children by Parents and Educators: A PRISMA Systematic Review

**DOI:** 10.1007/s10567-024-00506-2

**Published:** 2024-11-14

**Authors:** R. K. McLean, L. A. Tully, S. K. Brinley, T. Carl, A. Turnell, J. C. Northam, M. R. Dadds

**Affiliations:** 1 School of Psychology, Faculty of Science, University of Sydney, Sydney, NSW Australia; 2 School of Medicine and Psychology, The Australian National University, Canberra, Australia

**Keywords:** Child mental health, Preschool, Early learning, Universal screening, Multi-informant, Educator, Teacher, Parent, Universal screening, Universal mental health screening, Socio-emotional and behavioural wellbeing

## Abstract

Children develop rapidly in the preschool period, making accurate appraisals of mental health (MH) difficult. The preschool years are a key period for early identification of MH concerns and could benefit from multi-informant, universal MH screening (UMHS). This systematic review aimed to identify multi-informant UMHS measures for preschool-aged children, and to examine their clinical utility, effectiveness, and acceptability. Studies reporting the predictive and incremental validity, effectiveness or acceptability of parent and educator-report UMHS measures for children aged 3–5 years were identified through CINAHL, Embase, ERIC, Medline, PsycINFO, Scopus and Web of Science. Studies were excluded if screening was not the primary focus, not universal, single informant, or primarily focussed on Autism Spectrum Disorder. A total of 11 studies using 10 measures was identified. Ten studies screened for broad MH difficulties. Three educator-report and one parent- and educator-report measures had acceptable predictive validity. One study reporting incremental validity found that adding educator-report to parent ratings significantly improved the identification of MH concerns. No studies reported on effectiveness. Three studies that explored acceptability reported strong support for either UMHS in general or specific measures. There are promising results that UMHS can accurately identify child MH concerns in the preschool period using parent and educator reports. However, with few of the examined measures reaching the benchmark standards for predictive validity and only one study examining incremental validity, further research is needed to establish clinical utility. UMHS with preschool populations appears to be acceptable; future studies should further examine multi-informant screening in preschool populations. This systematic review was registered with the International Prospective Register of Systematic Reviews (PROSPERO; https://www.crd.york.ac.uk/prospero; registration number: CRD 42022383426).

**Supplementary Information:**

The online version contains supplementary material available at 10.1007/s10567-024-00506-2.

Children develop rapidly in the first 5 years of life and it is well established that many mental health (MH) difficulties first emerge during the preschool period from 3 to 5 years of age (Bayer et al., 2009; Lavigne et al., 2009; Oh et al., 2015; Robinson et al., 2008). Rapidly changing behaviour, social, and emotional development during this period of childhood can make accurate appraisals of child wellbeing difficult for parents (Konold et al., 2004). Parents and other caregivers of preschool-aged children, such as educators or teachers (hereafter referred to as educators), face challenges in distinguishing between normative behaviour and behaviour indicative of more serious concerns (Wakschlag et al., 2007). For example, behaviours such as tantrums, non-compliance and aggression can be age-appropriate and transitory, but may also be indicative of serious, disruptive behaviour problems, especially when these behaviours are severe and/or persist over time. Thus, primary caregivers such as parents and educators need tools to help them identify children who are at risk of developing MH difficulties, to increase access to early interventions. Universal mental health screening (UMHS) is a population-based approach in which individuals complete systematic assessments designed to identify those at risk of developing socio-emotional (internalising) and behavioural (externalising) difficulties (Humphrey & Wigelsworth, 2016). This review aims to examine the current literature that has examined UMHS measures in preschool children through multi-informant reports by parents and educators.

## Early Intervention and Identification in Children

Early intervention refers to a broad array of academic, medical or social support or treatment programs designed to enhance a young child’s development (Ramey & Ramey, 1998). Programs can be delivered to at-risk children, with the aim of being preventative, or targeted treatment programs implemented with children with known or diagnosed problems. Identifying children who may be at risk of MH difficulties, or recognising specific behaviour or emotional difficulties as requiring further investigation is the first step in early intervention. Early identification of children with MH concerns often leads to intervention, thus disrupting the often stable trajectory of MH difficulties from childhood to adulthood.

Parents and other primary caregivers play a significant role in help seeking for their children, that is, the recognition of emerging MH problems and facilitating access to services (Schnyder et al., 2020). The parental help-seeking process has been identified as (1) Parents’ recognition of MH difficulties, (2) Parents’ recognition of a need for professional help, (3) Parents’ actively seeking help, and (4) Family accessing and receiving necessary help/support (Reardon et al., 2017). However, parents do not always recognise behavioural or emotional difficulties as requiring further investigation, nor do they always appreciate the need for early intervention despite the established evidence base (Kowalenko, 2012). This is particularly pertinent in the preschool age range in which differentiating between psychopathology and normal early childhood development is a major challenge (Sim et al., 2019). A nationally representative survey of Australian parents of children under 18 years found that only 35% of parents were confident that they would recognise the signs of MH difficulties in their children and fewer than half (44%) knew where to access professional help if their child was experiencing difficulties, with confidence rates significantly lower in parents of younger than older children (Rhodes, 2017). A longitudinal study has shown that parents who recognise their child’s problem behaviour are more likely to access professional help for their child at age 3 (Oh et al., 2015). Thus, this evidence demonstrates the importance of recognising MH concerns as the first step towards parental help-seeking, including further assessment and accessing early intervention services.

Children with untreated MH difficulties can experience far-reaching negative effects throughout adolescence and into adulthood. In children as young as 3, behavioural and emotional difficulties have influenced wellbeing in later childhood and early adulthood across domains such as employment, education, criminal activity, physical and MH (Caspi et al., 1996; Jones et al., 2015). However, left untreated, the implications of low rates of identification mean that children in need of MH services frequently do not receive appropriate intervention and face poor outcomes.

Numerous papers have outlined the poor rates of help-seeking particularly amongst parents of young children (Ellingson et al., 2004), low rates of professional service utilisation amongst preschoolers with clinical diagnoses (Lavigne et al., 2009) or unmet MH needs in children more generally (Hiscock et al., 2020; Kataoka et al., 2002). In fact, the highest rates of unmet MH needs in the United States are in children under 6 years, who are also children of colour and from low-income families (Kataoka et al., 2002).

## Universal Mental Health Screening

Given overburdened healthcare systems and the long-term ramifications of untreated MH difficulties, UMHS offers a cost-effective mechanism for identifying young children in need of support (Humphrey & Wigelsworth, 2016). UMHS can be an effective tool for identifying MH concerns by improving recognition and increasing access to early intervention services, thereby facilitating help-seeking by parents or other informants (Humphrey & Wigelsworth, 2016). UMHS provides early identification of individuals with MH symptoms, who might require further follow up and it may also include referral pathways to appropriate services (Lavigne et al., 2016). It can be conducted as a single assessment with questionnaires which are scored according to clinical cut-offs, or involve multistage assessments in which children are identified in a first stage and receive further follow-up or assessment in subsequent stages. As with other standardised assessments, it can be completed individually by children, parents, educators, or other health professionals. UMHS does not make diagnoses of specific disorders and is not a diagnostic tool.

UMHS in the preschool years is important because it provides a critical opportunity to identify children at risk of MH difficulties and provide early intervention prior to difficulties becoming more entrenched and severe (Moore et al., 2022a). This is especially important since early intervention, implemented in early childhood, has demonstrated high rates of cost effectiveness (Heckman, 2008). Despite the potential benefits of UMHS for preschoolers, there is a paucity of research examining the efficacy, acceptability, and accuracy of UMHS in this developmental period (Anderson et al., 2019). A recent review by Becker-Haimes et al. (2020) of free, brief, and accessible child MH measures identified only two UMHS measures appropriate for use with preschool-aged children which were rated as having “excellent” psychometric accuracy, the *Pediatric Symptom Checklist* (PSC; Jellinek et al., 1988) and the *Strengths and Difficulties Questionnaire* (SDQ; Goodman & Goodman, 2009). Of these measures, only the SDQ was also identified as suitable for multi-informant use by parents and teachers. The current review builds on the work of Becker-Haimes et al. (2020) by specifically evaluating the effectiveness and acceptability of multi-informant screening measures in the preschool age range.

## Multi-Informant Screening

Multi-informant report is a hallmark of developmental research and practice as it captures information about the child from multiple sources. Therefore, it is likely that UMHS for preschoolers will be particularly effective when multiple reporters are included (Scott et al., 2011). Such approaches incorporate the unique perspectives of the child’s behaviour and wellbeing across settings such as the home and school or childcare by different caregivers (Anderson et al., 2019). Given the challenges of identifying normative versus concerning emotions and behaviour in the preschool-age bracket, secondary informants such as education or childcare providers are ideal as they may offer important and distinct insight into child MH in addition to parent perceptions (Feeney-Kettler et al., 2011; Smith, 2007). Educators are often familiar with children’s social interactions, behaviour and wellbeing as they spend significant amounts of time observing children in naturalistic environments and in play with similar-aged peers. In Australia and the United States, the majority of 4–5 year old children are enrolled in preschool or early childhood education and care, attending between 15–21 h per week on average (Australian Bureau of Statistics, 2021; National Center for Education Statistics, 2016, 2018), again emphasising that educators are well-placed to conduct UMHS with an accessible cohort of children (DeLucia et al., 2022). However, there remains a need for children with MH difficulties to be systematically identified and referred by educators as part of an integrated model for child MH assessment (Casale & Reyes, 2023).

In contrast to primary care settings or formal MH services, preschools, pre-kindergarten, childcare, long daycare or early learning centres (henceforth referred to as ‘preschools’) are an ideal setting for UMHS since they are less encumbered by issues of access and stigma (Desta et al., 2017). Like schools for older children, preschools offer a unique opportunity to address the MH needs of children (der Embse & De Los Reyes, 2024). Easy access to preschools means that many parents turn to educators or preschool staff in the first instance to discuss MH concerns they have about their child(ren). In fact, research shows that parents frequently seek help from an informal source such as educators, friends or family, prior to seeking help from professional medical specialists, education or MH services (Pavuluri et al., 1996). Yet preschool educators have been subject to minimal research regarding their perspectives on child MH, and their role in identifying and supporting young children with socio-emotional or behavioural concerns (Croft et al., 2015). Instead, research to date on educator involvement in UMHS has focussed on primary and secondary school teachers. Research with older children has shown that over-reliance on single sources of information and decision-making based on non-scientific factors (i.e., factors which are not evidence-based such as small standardisation samples which are not representative of the target population) contributes to inequitable school MH decision-making (von der Embse & De Los Reyes, 2024). These findings are likely to be relevant for younger children as well, therefore emphasising the need for validated, multi-informant assessment.

Identifying emotional and behavioural concerns in children can be undertaken with varying degrees of accuracy by parents and other primary caregivers each with their own level of biases, knowledge and understanding of the child. Whilst preschool educators have a referent classroom group by which to compare the child, they may have little to no experience in the area of child MH (Stormont & Stebbins, 2005). Screening practices that rely exclusively on either parent only or educator only reports omit important information about child behaviour in different settings and are at risk of misidentifying children with MH concerns (Eklund & Dowdy, 2014; Stefan & Miclea, 2017). Thus, in order to identify children who may require support and additional MH services, parents and educators need clinically useful and effective screening measures that allow for multi-informant reports to help them accurately identify children at risk.

## Clinical Utility and Effectiveness of Screening

A range of screening tools currently exist for identifying MH difficulties in young children; however, little is known about the multi-informant measures that exist, and the clinical utility and effectiveness of these measures. The clinical utility of these measures needs to be assessed to make value judgements of UMHS in this population group. However, “clinical utility” is a poorly defined construct in the UMHS literature and has been used to encompass an array of broad concepts such as feasibility, practicability, acceptability, perceived utility and treatment effectiveness (Murphy et al., 1996; Proctor et al., 2011; Schubiner et al., 1994). This review defines “clinical utility” as whether the intended screening outcomes have perceived clinical value, that is whether they are helpful and accurate in identifying children at risk of MH difficulties (Gall et al., 2000; Humphrey & Wigelsworth, 2016; Schubiner et al., 1994). Psychometric properties of screening measures can thus be used to assess the accuracy or validity of measures (Humphrey & Wigelsworth, 2016).

When screening tools are inaccurate and thus lack clinical utility, they can incorrectly categorise children as at-risk when in fact they are not, an error that is known as false positives. Over-identification and risks of high false-positives in UMHS can result in wasted resources where children are unnecessarily triaged to receive intervention and/or stigmatisation of children who do not require help (Sawyer et al., 2014). Conversely, under-identification in the form of failing to identify children who are in fact at-risk, the error of false negatives, carries the risk of denying children access to treatment that may assist them. Over- and under-identification can be measured by the psychometric properties of predictive validity, including specificity and sensitivity. Reviewing measurement properties can be a time-consuming task requiring an understanding of psychometrics. This review’s extraction of psychometric properties will, therefore, provide an accessible summary of the current literature.

In evaluating the clinical utility of multi-informant UMHS, the incremental validity of parent and educator report is of particular interest in considering the unique perspectives of each informant and what they add to an understanding of child MH. Adding educators’ perspectives to parent-report has the potential to increase the validity of early identification, whilst also raising the possibility of discrepant ratings between informants (Croft et al., 2015; De Los Reyes et al., 2015). The presence of multi-informant discrepancy has been well-reported and in fact offers important, domain-relevant information specific to each informant’s context e.g., home or school (De Los Reyes et al., 2023). However, the incremental, predictive validity of multi-informant approaches relative to the use of single informant report warrants further attention, especially in relation to young, preschool-age children.

Evaluating the effectiveness of an intervention can be assessed by its ability to achieve its intended outcomes (Andrews, 1999). More specifically, in the context of UMHS, effectiveness can be defined by the improvement of early identification of child MH concerns (Brinley et al., 2024). UMHS is thus considered effective if it improves the identification or diagnosis, referral, or treatment of child MH difficulties, which can be measured by rates of accurate identification of MH difficulties or risk, uptake of referral rates to MH services, or service uptake.

Whilst there are a number of previous reviews of screening by educators for Autism Spectrum Disorder in children, including preschool aged children (e.g., DeLucia et al., 2022), a recent systematic review examining the effectiveness and cost-effectiveness of school-based identification models including UMHS has found there is little research on the identification of MH difficulties in preschool-aged children (Anderson et al., 2019). As such there is a need to examine the literature regarding UMHS effectiveness in terms of service referral and uptake following identification.

## Acceptability of Screening

The acceptability of UMHS can impact uptake and implementation of screening in clinical and community settings (Harrison et al., 2013). It has been argued that UMHS in young children is inappropriate and ineffective, may increase parental anxiety, stigmatise children with labels, and lead to the over-medication of children (Frances, 2012; Jureidini & Raven, 2012). These arguments made against UMHS have in some instances halted the implementation of UMHS in preschool-age children previously (Alexander & Mazza, 2015). As such it is essential to investigate the perceived acceptability of UMHS amongst users if it is to be adopted and implemented widely as an early intervention strategy.

The current review is primarily interested in users’ perspectives of acceptability, that is, those of parents and educators. One element of acceptability comprises whether screening is considered appropriate, and/or perceived as helpful or useful. This may involve perceptions about whether the screening is wanted, needed or socially significant (Humphrey & Wigelsworth, 2016) or whether it is agreeable, palatable, or satisfactory (Proctor et al., 2011). Satisfaction can also encompass ‘usability’ of the tool (e.g., level of satisfaction with the length of screening, whether the language was easy to understand); whether informants felt comfortable or distressed by the screening; and whether informants would recommend screening to others, or if they would complete screening again in future (Brinley et al., 2024). Acceptability data, expressed as parental or educator attitudes towards screening, can be collected quantitatively or qualitatively. Importantly, acceptability encompasses characteristics that are associated with the likelihood of adoption of a screening measure (Glover & Albers, 2007; Kamphaus et al., 2007). This review will investigate the acceptability of UMHS broadly, and specific screening measures, as assessed by parents and educators.

No systematic review to date has evaluated multi-informant UMHS measures by parents and educators of preschool-aged children. As such, there is a need to identify what measures exist for children in this age range, examine the clinical utility and effectiveness of these measures, and also to examine the acceptability of UMHS more broadly.

## The Current Study

The aim of this systematic review is to examine multi-informant UMHS for preschool-aged children (3–5 years) by both parents and educators. The review will answer the following questions:

1. What are the existing MH screening measures utilised by both educators and parents of preschool-aged children and what is the clinical utility of these measures, that is, what is the predictive and incremental validity of these measures?
2. What is the effectiveness of UMHS?
3. What is the acceptability of utilising UMHS amongst educators and parents of preschool-aged children?

## Methods

The protocol for this systematic review was registered with the International prospective register of systematic reviews, PROSPERO, on 22 December, 2022 (Registration number CRD42022383426).

### Eligibility Criteria

Peer-reviewed studies examining UMHS for child mental health or socio-emotional and behavioural wellbeing in preschool children aged 3 years 0 months—5 years 11 months were included. Included studies reported universal screening whereby all children within a population-based sample undertook the systematic assessment of MH or socio-emotional or behavioural wellbeing and ratings were provided by both parents and educators.

Studies were excluded if they focussed specifically on Autism Spectrum Disorder or were not published in English. Complete inclusion and exclusion criteria are presented in Table 1.

**Table 1:** Inclusion and Exclusion Criteria

Inclusion criteria:	Exclusion criteria (in ranked order):
	Duplicate studies
Studies published in English	Studies not published in English
Studies published in peer-reviewed journals	Studies not published in peer-reviewed journals; grey or unpublished literature (dissertations, theses)
Full text available	No full text available
Empirical data presented	No empirical data presented (Systematic review/meta-analysis; Case study; Narrative review; Conference proceedings; Commentary; Protocol paper)
Studies focussed on individuals in the age range 3–5 years (mean age ≤ 3 years 0 months and ≥ 5 years 11 months, or more than half the sample within the age range)	Studies focussed on individuals outside of the age range 3–5 years (mean age ≤ 3 years 0 months and ≥ 5 years 11 months, or more than half the sample outside the age range). This includes excluding any studies in which:
	a. No age details for child samples are reported and individuals are not attending preschool or similar early childhood learning or childcare;
	b. No age details for child samples are reported and children are enrolled in kindergarten, elementary grade or above (e.g., grade one; secondary school, etc.);
	c. Sample (or sub-sample analyses) do not report mean and age range includes children under 2 years and 0 months or children over 6 years and 11 months
	Screening with wrong primary respondent such as Medical Professional (GP, Paediatrician, Nurse, etc.), Allied Health (OT, Social Work, etc.), Psychologist, or Child report
Screening with parent and educator report	Screening with parent report only or educator report only
Studies in which the primary focus is screening	Studies in which the primary focus is not screening (e.g., prevalence surveys)
Studies in which the screening is universally implemented	Studies in which the screening is not universally implemented (i.e., screening is implemented with targeted populations)
Screening with a primary focus on child mental health or socio-emotional and behavioural wellbeing	Screening with a primary focus other than child mental health or socio-emotional and behavioural wellbeing (e.g., parenting, parental mental health, physical health, academic performance)
	Studies which do not report predictive validity, incremental validity, effectiveness or acceptability data for screening measures
	Studies which focus on screening for Autism Spectrum Disorder

### Information Sources

Seven databases were searched for relevant literature: CINAHL, Embase, ERIC, Medline, PsycINFO, Scopus and Web of Science. All databases were searched via Ovid aside from CINAHL, Scopus and Web of Science.

### Search Strategy

The first author developed and translated the search strategy according to the relevant subject headings and functionality of each database in consultation with two authors (LT and MD) and a research librarian. The strategy was trialled in October and December 2022. Databases were searched in May 2023 using the following search strategy:

- 1. (exp mass screening/ or mass screening*.mp. or universal screening*.mp. or early intervention*.mp. or early identification*.mp. or screening*.mp. or exp needs assessment/ or needs assessment*.mp OR (mass adj3 screening*) OR (universal adj3 screening*)).
  AND
- 2. (exp mental health/ OR mental health.mp OR exp mental disorder/ OR wellbeing.mp OR wellbeing.mp OR well-being.mp OR ((mental or psychological or behavioral or socio-emotional or social?emotional or socioemotional) adj2 (disorder* or difficult* or health* or problem* or wellbeing or well-being)).mp).
  AND
- 3. (preschool student*.mp OR exp Child, Preschool/ OR preschool*.mp OR pre-kindergarten*.mp OR prekindergarten* OR early childhood*.mp OR early learning*.mp).
  AND
- 4. (exp school teachers/ OR teacher*.mp OR educator*.mp OR child care worker*.mp OR day care worker*.mp OR daycare worker*.mp OR childcare worker*.mp OR "prekindergarten teacher*" OR "pre-kindergarten teacher*").

Results were limited to peer reviewed journals.

To ensure literature saturation, we scanned the reference lists of included studies identified through the search and conducted manual handsearching.

### Study Selection

Article citations from the search results were uploaded to Covidence (Veritas Health Innovation). After duplicates were removed, the first author screened all papers by reviewing the title and abstract of papers and a team of five authors (SB, TC, RM, LT and AT) independently screened papers against criteria. A calibration exercise was undertaken to pilot and refine the screening questions. Studies not meeting criteria were excluded.

Full texts for the remaining studies were obtained and examined against criteria. Studies not meeting the criteria were excluded using a hierarchy of exclusion criteria as outlined in Table 1. All papers were screened by two reviewers to reduce selection bias. The first author screened all papers and the same team of five authors independently screened a selection of papers against criteria. At either stage of screening, a third reviewer was consulted when consensus could not be reached by prior discussion.

**Fig. 1:**
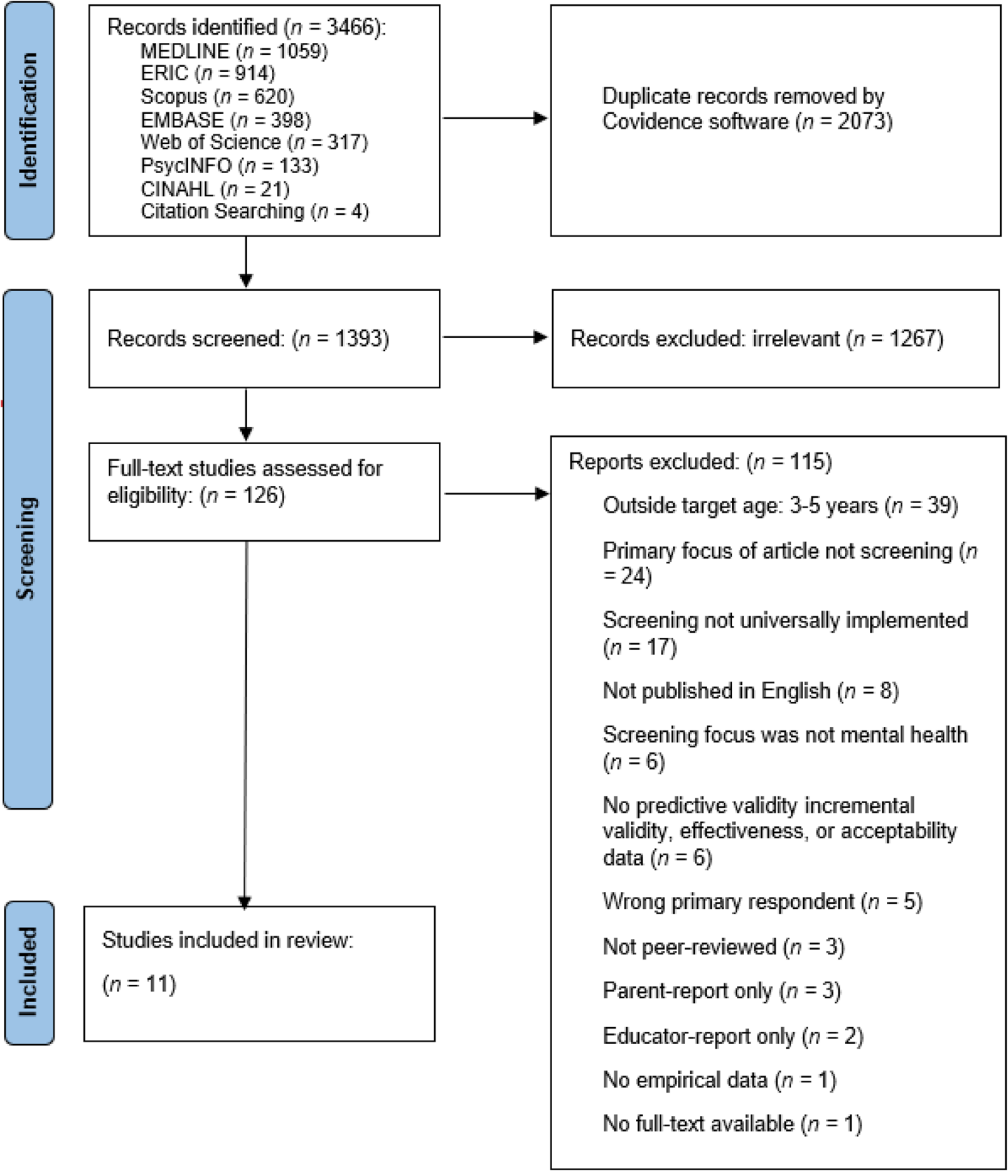
PRISMA Flow Diagram for Identification of Studies

The study selection process is outlined in Fig. 1.

### Data Collection

Data for the included studies were extracted in excel using a codebook developed by the authors. Six authors (SB, TC, RM, JN, LT and AT) independently conducted data extraction. Extraction into the codebook was trialled twice using two included studies. Codebook headings are included in Supplementary file 1. Our data collection only utilised published data and we did not contact study authors for additional information.

### Study Quality Assessment

All included studies were critically appraised for risk of bias using the Mixed Methods Appraisal Tool (MMAT) (Hong et al., 2018), an evaluation tool designed for systematic reviews that include mixed studies i.e., qualitative, quantitative or mixed methods studies. The MMAT comprises two stages. First, two initial screening questions confirm that the study is an empirical study and appropriate to be appraised by the MMAT. Second, for each included study, the appropriate methodological category (e.g., quantitative descriptive, quantitative non-randomised, qualitative) is selected before answering corresponding questions. A calibration exercise was undertaken to ensure all reviewers understood how to assess studies using the MMAT and resulted in an acceptable level of agreement between raters.

## Results

### Study Selection

Database searches yielded 3,466 results. After duplicates were removed, 1,393 were screened including 126 that underwent full-text review. The final number of studies included was 11. The study selection process is illustrated in Fig. 1.

### Study Characteristics

All studies were published within the past 10 years, aside from two studies published in 2007 (Barbarin) and 2011 (Feeney-Kettler et al.). Of the 11 studies included in the review, over half of the studies were conducted in the United States (*n* = 7). Studies were also conducted in Japan (*n* = 1), the Netherlands (*n* = 1), Romania (*n* = 1), and Spain (*n* = 1). There were no studies conducted in low and middle income countries.

Mental health domains screened in the included studies were mainly broad social, emotional, and behavioural wellbeing (*n* = 10), and ADHD (*n* = 1). All children were community samples recruited from either preschools or kindergartens (*n* = 8), community health clinics (*n* = 2) or random census sampling (*n* = 1). Of the studies reporting demographic details about parents, 70% of recruited samples were predominantly female. Of the studies that reported child gender (*n* = 6), all recruited samples were gender balanced i.e., between 45–55% female/male. No study reported non-binary gender for children. The socio-economic status and cultural diversity of samples varied across studies. A summary of study characteristics is provided in Table 2.

**Table 2:** Study Characteristics

Author (Year)	Country	Study Design	Population & Setting	Study Aims	Child Characteristics		
					Sample size (n)	Gender* (female, male)	Age range (M, SD)
Barbarin (2007)	USA	Cross-sectional analytical study	Study 1: Pre-K children in 40 randomly selected classrooms in 6 participating states Study 2: Children enrolled in the Head Start program or early childhood programs receiving partial state financial support	To determine the nature and prevalence of socio-emotional concerns parents and educators have about preschool children, the degree to which parents and educators agree on their concerns, and the psychometric properties of the ABLE	Study 1: 415 Study 2: 5,577	Study 1: NR Study 2: NR	Study 1: 4 yrs (NR) Study 2: 3–4 yrs (NR)
Doove et al., (2019)	Netherlands	Prospective observational study	Children aged 3 years at study entrance as part of the Monitoring Outcome Measurements (MOM) child development study	To define psychometric properties of the Dutch PEDS and three VAS about ‘parenting’, ‘child behaviour’ and ‘child competence’ at the age of 3 and 4 years	346	52%, 48%	3 years (3.0; 0.2)
Ezpeleta et al., (2013)	Spain	Cross-sectional analytical study	A random sample of 3-year-olds from the census of preschoolers in Barcelona	To evaluate the psychometric properties of the SDQ in sample of Spanish preschool children	Phase 1: 1,341 Phase 2: 622	Phase 1: NR, 50.9% Phase 2: NR, 50%	Phase 1: 3 years (3.0; 0.18) Phase 2: 3 years (3.0, 0.16)
Feeney-Kettler et al., (2011)	USA	Quantitative Descriptive	Children in *n* = 22 preschools and childcare facilities in Southern California and Tennessee	To streamline the PBSS Phase 2 to create separate parent & educator versions, and to evaluate psychometric properties of the PBSS as a cost-efficient universal screening tool	NA	49%, 51%	3–5 years (NR)
Feeney-Kettler et al., (2019)	USA	Quantitative Descriptive	Preschool Children recruited from daycares and preschools (number not reported) in a large southern city and its surrounding suburbs in the US	To evaluate the multiple-gate PBSS for identifying children’s social, emotional, and behavioral difficulties	122	48%, 44%	3–5 years (NR)
Girio-Herrera et al., (2015)	USA	Cross-sectional analytical study	Kindergarteners at *n* = 18 elementary schools (Study 1) *n* = 5 elementary schools (Study 2)	To examine the IRS as a screening tool for detecting kindergarten children who are at risk for social, emotional, academic, and behavioral difficulties	NA	Study 1: NR, 46.8% Study 2: NR, 50.8%	5 years 1 month–5 years 11 months Study 1: (5.48, 0.32) Study 2: (5.61, 0.46)
Kettler et al., (2017)	USA	Cross-sectional analytical study	Preschool children taken from a convenience sample of *n* = 33 preschools in a heavily populated Northeastern US state	To determine the internal consistency, cross-informant agreement of a multi-gate screener and the concurrent relations among scores from single-gate and multi-gate screeners	105	NR, 60%	3–5 years (NR)
Moore et al. (2022a)	USA	Cross-sectional analytic and longitudinal cohort study	Preschool children from *n* = 5 state-funded preschools from a Title 1 (low income supported) school district	To examine the use of informants at the preschool level when universally screening for behavioral and emotional risk	535	48.3%, 44%	3–5 years (4.32, NR)
Moore et al. (2022b)	USA	Quantitative Descriptive	Preschool, Kindergarten, and first-grade children from *n* = 5 state-funded preschools housed within public elementary schools	A preliminary examination of educators’ and parents’ beliefs related to their participation in Universal Mental Health Screening	NA	NR, NR	NR (NR)
Stefan et al., (2017)	Romania	Cross-sectional analytical study	Preschool children from *n* = 3 preschools within an urban area of Cluj-Napoca	To evaluate the reliability and validity of the ECS and SCS parent and educator versions	Sample 1: 107 Sample 2: 73	Sample 1: 48.6%, 51.4% Sample 2: 54.8%, 45.2%	Sample 1: 2 years, 4 months–4 years (43.84 months, NR) Sample 2: 4–5 years (4.33, NR)
Takayanagi et al., (2016)	Japan	Quantitative Descriptive	Five-year-old children from a local community health check-up run through municipal health centres in Aomori prefecture	To verify the psychometric properties of the ADHD-RS in identifying preschool children with ADHD using DSM-5 criteria at a community health check-up for five-year-olds	838	46.1%, 53.9%	4 years. 10 months–5 years, 10 months (4.83, 0.28)

*no studies reported non-binary child gender. *ABLE* Attention Behaviour Language Emotions, *ADHD* Attention-Deficit/Hyperactivity Disorder, *ADHD-RS* Attention-Deficit/Hyperactivity Disorder Rating Scale, *DSM-5* Diagnostic and Statistical Manual of Mental Disorders, Fifth Edition, *ECS* Emotion Competence Screening, *IRS* Impairment Rating Scale, *NR* not reported, *PBSS* Preschool Behavior Screening System, *PEDS* Parents’ Evaluation of Developmental Status, *SCS* Social Competence Screening, *US* United States, *VAS* Visual Analogue Scales

### Results of Individual Studies

#### Overview of Measures

The research studies examined 10 measures. As is expected for universal screening measures of community populations, almost all measures assessed broad constructs of internalising and externalising difficulties, including the *Attention, Behaviour, Language, Emotions* (ABLE; Barbarin, 2007); *Behaviour Assessment System for Children* (BASC; Moore et al., 2022a, 2022b); *Behavior Assessment System for Children—Behavioral and Emotional Screening System*, (BASC-2—BESS; Kettler et al., 2017); *Emotion Competence Screening* (ECS; Stefan & Miclea, 2017); *Impairment Rating Scale* (IRS; Girio-Herrera et al., 2015); *Parents’ Evaluation of Developmental Status* (PEDS; Doove et al., 2019); *Pediatric Symptom Checklist-17* (PSC-17; Moore et al., 2022a); *Preschool Behavior Screening System* (PBSS; Feeney-Kettler et al., 2011, 2019; Kettler et al., 2017); *and Strengths and Difficulties Questionnaire* (SDQ; Ezpeleta et al., 2013). One measure focussed on attention or hyperactivity, *ADHD-Rating Scale-IV* (Takayanagi et al., 2016). Table 3 details the measures used in the studies. Some measures also included prosocial or adaptive behaviour (ECS, IRS, PBSS, SDQ); and the PEDS measured developmental domains in addition to social, emotional and behavioural constructs (Doove et al., 2019). The number of items in each screening measure varied widely, particularly as some measures were multiphasic and included additional items dependent on previous responses (e.g., for each issue that a respondent indicated concern, follow-up questions were then asked about the level of severity and impact on the child’s life).

**Table 3:** Screening Measure Properties

Author (Year)	Sample N (Educator, Parent)	Screening measure (no. of items)	Mental health domain	Predictive validity				
				Criterion measure	Sensitivity (Educator, Parent)	Specificity (Educator, Parent)	PPV (Educator, Parent)	NPV (Educator, Parent)
Barbarin (2007)	Study 1: 238, 415 Study 2: NR, NR	ABLE (Stage 1: 10, Stage 2: 40)	Attention, Behaviour, Language, Emotions	Study 1 TCRS; BPI; ORCB; PPVT; OWLS Study 2: N/A	**Educator Pre-K**: 0.42 **K:** 0.65 **Parents:** 0.59 (end of Pre-K) 0.60 (end of K)	**Parent:** good .86 (end of pre-K), .84 (end of K)	NR NR	NR NR
Doove et al., (2019)	294, 329	VAS (3) PEDS– Dutch Version (10)	School Readiness/ Social Participation	CBCL SDQ	**Educator:** Child competence VAS 82.8 PEDS: 96.6 **Parent:** Parenting VAS: 90.9 Child behaviour VAS: 71.4 PEDS: 80.6	**Educator** Child competence VAS 82.8 PEDS: 83.1 **Parent:** Parenting VAS: 78.4 Child behaviour VAS 80.8 PEDS: 80.2	NR NR	**Educator** Child competence VAS 97.6 PEDS: 99.5 **Parent** Parenting VAS: 99.2 Child behaviour VAS 95.9 PEDS: 98.9
Ezpeleta et al., (2013)	**Phase 1** N/A, 1,341 **Phase 2** 94, 622	SDQ (25)	Behaviour Difficulties	CBCL (1.5—5 years); CGAS; DICA-PPYC; SDQ (Spanish and Catalan versions)	**Educator** Screening cutoff: 72.1 Borderline: 38.2 Abnormal: 18.5 **Parent** Screening cutoff: 74.2 Borderline: 52.1 Abnormal: 29.9	**Educator** Screening cutoff: 44.3 Borderline: 78.3 Abnormal: 89.2 **Parent** Screening cutoff: 62.1 Borderline: 83.4 Abnormal: 95.8	NR NR	NR NR
Feeney-Kettler et al., (2011)	112 113	PBSS (46)	Internalising Externalising Prosocial Behaviour	BASC-2	**Educator Tot** = .94 Int = .96 Ext = 1.00 **Combined Ed/P** Tot = .98 Int = .89 Ext = .98 **Parent:** Tot = .96; Int = .80 Ext = .90	**Educator Tot** = .51 Int = .59 Ext = .71 **Combined Ed/P** Tot = .28 Int = .42 Ext = .48 **Parent:** Tot = .49 Int = .61 Ext = .59	**Educator Tot** = .49 Int = .44 Ext = .54 **Combined Ed/P** Tot = .57 Int = .62 Ext = .57 **Parent:** Tot = .39 Int = .57 Ext = .51	**Educator Tot** = .95 Int = .98 Ext = 1.00 **Combined Ed/P** Tot = .93 Int = .77 Ext = .97 **Parent:** Tot = .96 Int = .82 Ext = .93
Feeney-Kettler et al., (2019)	122 122	PBSS (46)	Internalising, Externalising, Prosocial Behaviour	BASC-2	NA NA	NA NA	NA NA	NA NA
Girio-Herrera et al., (2015)	**Study 1:** 56 12 **Study 2:** 568 273	IRS (31)	**Study 1**: Academic, Social, Behaviour, Family **Study 2:** Academic, Social, Behaviour, Family	**Study 1:** BASC-2 **Study 2:** BESS	**Study 1:** **Educator** 0.57 **Parent** 0.14 **Study 2:** **Educator** 0.72 **Parent** 0.17	**Study 1:** **Educator** 0.92 **Parent** 0.98 **Study 2:** **Educator** 0.95 **Parent** 0.95	**Study 1:** **Educator** 0.65 **Parent** 0.98 **Study 2:** **Educator** 0.65 **Parent** 0.95	**Study 1:** **Educator** 0.89 **Parent** 0.74 **Study 2:** **Educator** 0.97 **Parent** 0.90
Kettler et al., (2017)	105 105	PBSS (46)	Social, Emotional, and Behaviour Difficulties	BASC-2, C-TRF, CBCL	**P1 + P2** **Educator** ASEBA TP Borderline = 0.76, Clinical = 0.97 **Parent** ASEBA TP Borderline = 0.66, Clinical = 0.90	**P1 + P2** **Educator** ASEBA TP Borderline = 0.96, Clinical = 0.91 **Parent** ASEBA TP Borderline = 0.91, Clinical = 0.90	**P1/P2** **Educator** ASEBA TP Borderline = 0.80, Clinical = 0.45 **Parent** ASEBA TP Borderline = 0.91, Clinical = 0.90	**P1/P2** **Educator** ASEBA TP Borderline = 0.95, Clinical = 1.00 **Parent** ASEBA TP Borderline = 0.95, Clinical = 0.99
Moore et al. (2022a)	14 535	BASC-3 BESS (86); PSC-17 (17)	Behavioural and Emotional Risk	BASC-3 BESS	NR NR	NR NR	NR NR	NR NR
Moore et al. (2022b)	40 330	BASC-3 BESS (86)	Behavioural and Emotional Risk	PSC-17	NA NA	NA NA	NA NA	NA NA
Stefan et al., (2017)	NR 180	ECS (30)	Social and Emotional Competencies	SRSS	**Educator** ECS-T to C-TRF: Sensitivity indices between 70.00—75.00 (internalising), and 75.00—83.33 (externalising) SCS- T to C-TRF: 75.00 to 77.78 (internalising); and 71.43- 75.00 (externalising) **Parent** ECS-P to CBCL: 80.00—84.62 (internalising); and 69.23—71.43 (externalising) SCS- P to CBCL: 76.92—80.00 (internalising); and 71.43—75.00 (externalising)	**Educator** ECS-T to C-TRF: 92.78–93.85 (internalising). and 92.21—94.74 (externalising) SCS-T to C-TRF: 91.84–94.20 (internalising), and 90.79—91.58 (externalising) **Parent** ECS-P to CBCL: 92.65—93.62 (internalising), and 92.42%—92.47 (externalising) SCS- P to CBCL: 89.36—92.65 (internalising), and 88.42%—93.94 (externalising)	**Educator** ECS-T to C-TRF: 50.00–60.00 (internalising); 45.45–64.29 (externalising) SCS-T to C-TRF: 42.86–46.67 (internalising); 41.67–52.94 (externalising) **Parent** ECS-P to CBCL: 44.44–64.71; (internalising); 50.00–56.25 (externalising) SCS-P to CBCL: 44.44–50.00 (internalising); 45.00–55.56 (externalising)	**Educator** ECS-T to C-TRF: 96.53–96.77 (internalising); 96.77–98.61 (externalising) SCS-T to C-TRF: 97.83–98.48 (internalising); 96.67–97.18 (externalising) **Parent** ECS-P to CBCL: 97.78–98.44 (internalising); 95.56–96.83 (externalising) SCS-P to CBCL: 96.55–98.44 (internalising); and 96.55–96.88 (externalising)
Takayanagi et al., (2016)	NR 838	ADHD-Rating Scale-IV Parent (18); Educator (18)	ADHD	SDQ	**Educator** Above 90th percentile 30.23 **Parent** Above 90th percentile 89.13	**Educator** 90.92 **Parent** 94.07	**Educator** 16.05 **Parent** 46.59	**Educator** 95.78 **Parent** 99.33

Data reported for recommended cutoff scores or best tradeoff between sensitivity and specificity. *ABLE* Attention Behaviour Language Emotions, *ADHD* Attention-Deficit/Hyperactivity Disorder; *ASEBA* Achenbach System for Empirically Based Assessment, *BASC-2/3* Behaviour Assessment System for Children Second/Third edition, *BESS* Behavioral and Emotional Screening System, *BPI* Behaviour Problem Index, *CBCL* Child Behavior Checklist, *CGAS* Children’s Global Assessment Scale, C-TRF Caregiver-Teacher Rating Form, *DICA-PPYC* Diagnostic Interview for Children and Adolescents for Parents of Preschool And Young Children, *ECS-P/T* Emotion Competence Screening Parent/Teacher Form, *Ext* externalizing, *K* Kindergarten, *Int* internalizing, *IRS* Impairment Rating Scale, *NR* not reported, *ORCB* Observer ratings of Child Behaviour, *OWLS* Oral and Written Language Scale, *PBSS* Preschool Behavior Screening System, *PEDS* Parents’ Evaluation of Developmental Status, *PPVT* Peabody Picture Vocabulary Test, *PSC-17* Pediatric Symptom Checklist-17, *SCS-P/T* Social Competence Screening Parent/Teacher Form, *SDQ* Strengths and Difficulties Questionnaire, *SRSS* Social Skills Rating System, *T* total problems, *TCRS* Teacher Child Rating Scale, *VAS* Visual Analogue Scales

Most studies reported inter-rater reliability between parent and educator informants using Pearson’s correlations. Two studies reported moderate cross-informant agreement between parent and educator ratings (Barbarin, 2007; Feeney-Kettler et al., 2011). Barbarin (2007) reported that parents and educators agreed 77% of the time about children who did not have difficulties. One study presented chi-square analyses of cross-informant agreement, reporting a statistically significant association between parental concerns and professional caregivers’ concerns about child wellbeing and development at baseline and 10-month follow-up (*χ*^*2*^ = 34.8; df = 1, *p* < 0.001 and *χ*^*2*^ = 8.1; df = 1, *p* = 0.004) (Doove et al., 2019).

The majority of studies (*n* = 7) that reported internal consistency for parent-report measures reported high reliability (Cronbach’s alphas above 0.8) (Ezpeleta et al., 2013; Feeney-Kettler et al., 2011, 2019; Kettler et al., 2017; Moore et al., 2022a; Stefan & Miclea, 2017; Takayanagi et al., 2016). Most studies (*n* = 7) that reported internal consistency for educator-report measures reported high or very high reliability (Cronbach’s alphas above 0.8) (Ezpeleta et al., 2013; Feeney-Kettler et al., 2011, 2019; Kettler et al., 2017; Moore et al., 2022a; Stefan & Miclea, 2017; Takayanagi et al., 2016). Three studies did not report internal consistency for either parent or educator report (Barbarin, 2007; Girio-Herrera et al., 2015; Moore et al., 2022b).

Few studies reported test–retest reliability (Doove et al., 2019; Feeney-Kettler et al., 2011; Stefan & Miclea, 2017). Of the three studies that did, all reported good to excellent reliability for parent report and three studies reported good to excellent reliability for educator report. See Supplementary file 1 for further details regarding screening measures’ inter-rater reliability, internal consistency, and test–retest reliability.

#### Clinical Utility

To assess clinical utility, the predictive and incremental validity of measures were extracted. Studies utilised a range of criterion measures to test the predictive validity of their screening measures. Criterion measures varied widely with only one study utilising gold standard diagnostic interviews (Ezpeleta et al., 2013) or comprehensive assessments (*n* = 6) such as the Child Behavior Checklist (CBCL;Achenbach, 1999) or Caregiver-Teacher Rating Form (C-TRF; Achenbach & Rescorla, 2000), whilst others utilised other brief screening measures such as the PSC-17 or SDQ (*n* = 5) or a combination of measures. See Table 3 for details.

Seven out of the eight studies examining predictive validity reported acceptable or good sensitivity or specificity for parent ratings. Kettler et al. (2017) reported the strongest predictive validity for children in the clinical range: sensitivity 90%, specificity 90%, Positive Predictive Value (PPV) 42% and Negative Predictive Value (NPV) 99%. This study tested the PBSS against a comprehensive range of criterion measures including the BASC-2—BESS, C-TRF and CBCL 1.5–5, which included both parent and educator reports.

Kettler et al. (2017) also reported the strongest predictive validity for educator ratings for children in the clinical range: sensitivity 97%, specificity 91%, PPV 45% and NPV 100%. When compared to the criterion measure, educators were slightly more accurate raters than parents.

The sensitivity of the SDQ was poor for both parent and educator reports, across all subscales aside from the prosocial subscale (Ezpeleta et al., 2013). One study reported combined predictive validity for parent and an educator reports incorporating multi-informant reports to identify children at risk of internalising and externalising difficulties, with the PBSS showing combined sensitivity 98% and specificity 28% (Feeney-Kettler et al., 2011). Three studies did not report predictive validity (Feeney-Kettler et al., 2019; Moore et al., 2022a, 2022b).

The majority of studies (*n* = 8) reported Area Under Receiver Operating Characteristic Curve (AUC) analyses as an indicator of screening accuracy. Criterion measures again varied widely as did the level of analyses conducted (e.g., informant report, subscale, child gender, etc.). All studies reporting AUC analyses for parent report (*n* = 7), reported fair (above 0.70) to excellent (0.90–1.00) AUCs. Five studies reporting AUC analyses for educator report, reported very good to excellent AUCs (0.80–1.00) (Doove et al., 2019; Feeney-Kettler et al., 2011, 2019; Kettler et al., 2017; Stefan & Miclea, 2017). Two studies reported AUCs that were below acceptable levels for educator report (Ezpeleta et al., 2013; Takayanagi et al., 2016). Finally, only one study (Girio-Herrera et al., 2015) tested cross-informant ratings by parent-reported Impairment Rating System (IRS) identifying educator-rated BASC 2 at-risk status, and educator IRS identifying parent-based BASC 2 at-risk status—both resulting in poor AUCs.

#### Incremental Validity

Only one study reported the incremental validity of parent and educator reports in terms of child social-emotional outcomes (Moore et al., 2022a). Using hierarchical regression modelling, this study reported that educator-report of social-emotional difficulties using the BASC-3 BESS *Behavioural and Emotional Risk Index* (BERI) was significantly associated with kindergarten social-emotional readiness (*β* = − 0.46), however including parent-report BESS BERI did not significantly improve prediction of kindergarten social-emotional readiness. However, when parent-report was entered in the first block, parent-report was significantly associated with kindergarten social-emotional readiness (*β* = − 0.19) and when educator ratings were added in the second block, educator-report was also significantly associated with kindergarten social-emotional readiness (*β* = − 0.44) although parent ratings in the second block were no longer significantly related to readiness (*β* = − 0.07, *p* = 0.26).

In addition, Moore et al. (2022a) reported incremental validity using a second screening measure, the PSC-17. Educator-reported social-emotional difficulties using the PSC-17 was significantly associated with kindergarten social-emotional readiness (*β* = − 0.43) and parent report was not associated with kindergarten social-emotional readiness nor did inclusion result in significant improvements in variance in the model (*β* = 0.05, *R*^2^ = 0). When the parent report was entered in the first block, it was not statistically significant. However, when educator ratings were added to subsequent blocks, educator ratings were significantly associated with kindergarten social-emotional readiness (*β* = − 0.44) and parent ratings were no longer significantly related to social-emotional readiness. It is important to note that kindergarten social-emotional readiness is a criterion variable that references child behaviour in the education context only and therefore criterion contamination (whereby differences in parent and educator ratings may have arisen due to educators’ influence over the criterion variable, in this case social-emotional readiness) may be a factor in these findings which seemingly favour educator report. As Moore et al. (2022a) conclude, both parents and educators offer valuable information when screening of a child’s MH risk. However, educator report may be more informative than a parent report for predicting kindergarten social-emotional readiness, which we note is an education-specific measure of MH. It is also important to note that this study did not report other psychometrics of interest such as predictive validity more generally.

#### Effectiveness

Only one study reported the effectiveness of the screening measure in terms of referral uptake and longitudinal outcomes for children after screening preschool children using the Attention, Behavior, Language, and Emotions (ABLE) (Barbarin, 2007). However, this study only focussed on parent reports, and did not include the effectiveness of educator reports or multi-informant data. Moreover, this study reported effectiveness in terms of referral for Individualised Education Plans (IEP), the same outcome used to test predictive validity of the ABLE. After conducting screening for preschool children, 13.1% of children who were identified as having serious concerns through parent-reported ABLE were referred for IEP by the end of kindergarten. This was compared to 3.7% of children for whom no concerns were identified. The study did not report if this was a significant difference. The remaining studies did not report effectiveness data about screening measures.

#### Acceptability of UMHS

Only three studies examined the acceptability of screening measures or UMHS in general amongst parents and educators. Two studies investigating the acceptability of the PBSS measure found that 88% of parents in one sample (*n* = 107) and 87% of parents in another sample (*n* = 91) reported that the PBSS could provide useful information about their child, and 91% (*n* = 111) and 94% (*n* = 99) found it clearly written (Feeney-Kettler et al., 2019; Kettler et al., 2017). Amongst educators, 88% (*n* = 107) and 71% (*n* = 74) indicated that the PBSS could provide useful information about preschool children, and 89% (*n* = 109) and 88% (*n* = 83) found it clearly written (Feeney-Kettler et al., 2019; Kettler et al., 2017).

The initial study reported that just over half of educators (55%, *n* = 58) would be likely to use the PBSS to screen all their students for social and emotional wellbeing and some educators indicated that they would select students to screen rather than implement screening universally (Kettler et al., 2017). The second study reported that 71% of educators (*n* = 87) indicated that they would be likely to use the PBSS to screen all students (Feeney-Kettler et al., 2019). Neither study reported the number of items in the acceptability or evaluation measure.

A third study measured acceptability of UMHS more generally using four items encompassing constructs of importance, usefulness, willingness and appropriateness (Moore et al., 2022b). On average, educators and parents agreed or strongly agreed that it is important for schools to ask questions about child MH, screening is useful for identifying at-risk children, and MH development should be addressed in school (Moore et al., 2022b). Only 0.3–1.8% (*n* = 6) of parents and 0–10.0% (*n* = 4) of educators disagreed or strongly disagreed with the acceptability of UMHS (Moore et al., 2022b).

#### Risk of Bias in Studies

All studies were assessed using the MMAT (Hong et al., 2018). All studies were considered moderate to high quality. Quantitative descriptive studies tended to rate poorly for the risk of non-response bias or explaining non-responders and low response rates. Non-randomised trials frequently did not adequately account for confounders in the design or analysis. There were no randomised trials identified.

## Discussion

The aim of this review was to systematically examine research conducted on multi-informant UMHS for preschool-aged children by both parents and educators. We identified which measures have been utilised in the preschool population and examined their clinical utility in terms of predictive and incremental validity and effectiveness. The review also examined the acceptability of these measures amongst parents and educators. The recency of included studies suggests that this is an emerging area of research, conducted mainly in the United States and in wealthy, industrialised nations. Identified screening measures were highly varied and for some aspects examined, such as incremental validity, effectiveness and acceptability, there was a paucity of research making definitive conclusions difficult. However, there were 11 included studies and 10 measures identified for multi-informant UMHS in the preschool period, with promising findings for some measures suggesting that UMHS can accurately identify child MH concerns using parent and educator reports.

In order to assess the clinical utility of screening, this review examined the predictive and incremental validity of MH screening measures. Several screening measures demonstrated strong predictive validity for identifying children at-risk of MH difficulties. However, as is common for predictive validity of screening measures, several studies reported either high specificity or high sensitivity due to the tendency of sensitivity and specificity to be inversely related, and the challenge of developing a measure with maximal precision. Interestingly, the research showed that more educator-report measures had strong predictive validity (Girio-Herrera et al., 2015; Kettler et al., 2017; Stefan & Miclea, 2017) compared to parent-report (Kettler et al., 2017; Takayanagi et al., 2016). Using the benchmark of at least 70% sensitivity, 70% specificity and 50% PPV for developmental screening measures (Aylward, 1997), the ECS, IRS and PBSS met these standards for educator ratings (Girio-Herrera et al., 2015; Kettler et al., 2017; Stefan & Miclea, 2017). On the other hand, only the PBSS met this benchmark for parent-report (Kettler et al., 2017). However, results for this measure were mixed as earlier studies found that the combined, multi-informant predictive validity for the PBSS did not meet standards (Feeney-Kettler et al., 2011).

Surprisingly, the preschool version of the widely used SDQ demonstrated poor predictive validity with low sensitivity across both parent and educator reports and borderline acceptable to good specificity for parents and educators (Ezpeleta et al., 2013). Only the hyperactivity subscale had acceptable sensitivity and specificity at the recommended cut points and the study reported poor educator-report screening accuracy in terms of AUC analysis. Other comprehensive reviews of the SDQ as a screening measure amongst preschool populations have found a number of problems with its predictive validity, internal consistency of its subscales, test–retest reliability and criterion (concurrent) validity and have cautioned its use within non-clinical populations (Kersten et al., 2016; Lavigne et al., 2016). The preschool version of the SDQ may not have strong clinical utility or other psychometric properties as it has not been specifically developed for preschool samples, but further research is required to investigate this.

Only one included study reported on the incremental validity of adding educators’ ratings to parent ratings, finding a significant improvement in the identification of socio-emotional outcomes over time (Moore et al., 2022a). Contrary to previous research with older children, this study found that parent ratings did not significantly improve the prediction of outcomes. Previous findings with older children reported that parent ratings of preschool children added significant variance to child outcomes when considered in addition to later kindergarten teacher ratings (Owens et al., 2015) and a large, longitudinal study of children aged 4–11 years highlighted the power of combining informant reports to predict child outcomes across a number of domains (Verhulst et al., 1994). When evaluating the predictive and incremental validity of these measures, it is important to consider the criterion variables used in each analysis, and the context in which they reference child behaviour, such as the home, education or care setting. When criterion measures are mapped across multiple settings, it is easier to achieve the ideal model of child assessment in which child wellbeing is captured from multiple perspectives (De Los Reyes et al., 2023; Makol et al., 2020). There is promising work emerging which examines the use of screening measures with preschool children through parent, educator and child psychologists’ ratings, highlighting the value of gaining multi-faceted assessments of child wellbeing, but there remains an opportunity to research this further (Gustafsson & Sund Levander, 2024).

These results contribute to the evidence that whilst educators often have minimal MH training, their knowledge of a referent classroom group of children may be advantageous in their assessment of child wellbeing and development, given strong clinical utility for educator-report. Future research should focus on developing and evaluating UMHS measures, with high screening accuracy and clinical utility for use in preschool populations by educators.

Examining the effectiveness of UMHS adds to the weight of evidence for multi-informant screening because in the context of UMHS, improved identification is clearly linked to increased diagnosis, referral and/or treatment and thus, improved outcomes for children. Yet our review found no studies reporting on the effectiveness of multi-informant raters. This follows a consistent pattern of omission in the literature in which effectiveness data, in terms of screening outcomes or the consequences of screening, are rarely reported adequately or at all (Houri & Miller, 2020). There is a distinct lack of data reporting screening effectiveness and limited research measuring the effectiveness of UMHS in the child MH space (Brinley et al., 2024). In the case of screening amongst preschool children, none of the included studies investigated the effectiveness of multi-informant screening. This paucity of research remains despite multiple calls that we need to better understand the outcomes and consequences of screening. By effectively understanding outcomes, we can better understand the impact of UMHS (Houri & Miller, 2020). Thus, there is a clear need for future research to examine the effectiveness of screening in preschool populations with additional longitudinal studies measuring MH outcomes.

Given multi-informant report has long been the hallmark of developmental research and despite previous calls to action from highly cited research (e.g.,Achenbach et al., 1987; De Los Reyes et al., 2015), there remains a substantial gap in the study of multi-informant UMHS measures. There is a clear need for more research to demonstrate the incremental value of combining parent and educator reports in UMHS. Given demands on educators’ and parents’ time, lack of MH training for preschool educators and limited MH resources available to educators, the value of combined contributions needs to be made explicit. By demonstrating the predictive power of multi-informant reports, researchers can build the case for UMHS and improve child socio-emotional outcomes.

Critics have raised concerns that UMHS is not warranted in young children as it stigmatises them (Frances, 2012; Jureidini & Raven, 2012), and is neither wanted by parents nor educators (Humphrey & Wigelsworth, 2016). However, there is a mounting case refuting these conjectures. All three studies included in this review that investigated acceptability of UMHS reported strong support from parents and educators, for either specific screening measures or UMHS more generally in the preschool-age population. Importantly, each of these respondents evaluated the acceptability of UMHS after having completed screening for their children. A recent review of parent UMHS for children found that parents generally regarded UMHS as appropriate, across a range of contexts including schools, paediatric hospital settings, and well-child visits, although not all studies required parents to actually complete UMHS for their child (Brinley et al., 2024). The findings of the current review, therefore, add to existing research indicating that UMHS is, in fact, considered highly acceptable. Overall, the positive support from parents and educators for screening provides further evidence for the contention that preschools are ideal settings for screening, as they are accessible, less encumbered by MH stigma and a natural fit given parents frequently turn to educators for MH advice for their children (Desta et al., 2017; Pavuluri et al., 1996).

Whilst the studies in this review largely focussed on the acceptability of specific measures, there is a need to review screening more generally and in the current context. It may be possible that parents and educators have a growing awareness and understanding of early intervention for supporting child wellbeing, particularly in the aftermath of the global pandemic that had and continues to have significant ramifications on the wellbeing of children. More qualitative and quantitative research should be conducted in this area to identify perceived barriers, facilitators and perceived acceptability of multi-informant screening of preschool-aged children.

## Strengths and Limitations

This review was the first to identify multi-informant UMHS measures utilised by parents and educators in preschool child populations and the first to evaluate their clinical utility, effectiveness and acceptability. However, there are a number of limitations that need to be kept in mind when interpreting the results. Studies that were excluded from this review included those that were not published in English, did not report predictive validity, effectiveness or acceptability data or were not peer reviewed. The exclusion criteria for child age may have limited the results and inadvertently excluded studies that included preschool-aged populations. It was often difficult to discern if 3–5 year old children were included in studies with broad-age range samples. The ages of “preschool” children were not always specified and differed between states or countries, as the age of school entry differed across jurisdictions. Secondary source or missing data were not sought from any authors to provide clarification. Moreover, some aspects of the reviewed literature were difficult to synthesise because of the disparate measures utilised. Many studies reported on author-developed measures, with varying identification methods, scoring and subscale outcomes, making synthesis difficult. Finally, risk of bias presents a possible limitation in the area of recruitment bias across the included studies. In evaluating the methodological quality of included studies using the MMAT, some studies may have been biased by not recruiting a representative sample of the target population (Hong et al., 2018). Given UMHS is intended for use amongst general, non-clinical populations in the community, it is of utmost importance that screening measures are tested in samples which are as representative as possible. The authors suggest that recruitment methodology should match implementation of UMHS in practice in order to minimise confounding variables in testing the efficacy of universal screening of whole populations. For example, UMHS measures should be implemented universally with as few participant eligibility criteria as reasonable and without interference from non-systematic factors such as teacher-nomination.

## Implications

Parents and educators of preschool-aged children are integral to facilitating help-seeking for young children in need of MH support. They have a central role in early identification of MH problems and are a primary part of the pathway to enabling a child to access healthcare practitioners (De Los Reyes et al., 2015). However, based on the findings of this review, there are few measures that can be recommended to professionals in preschool and early childhood health settings for use with preschool-aged children. Based on the clinical utility of the included measures, the PBSS was the only measure to reach benchmark standards for predictive validity for both educator and parent report, however, the measure has not been published or made publicly available (Kettler et al., 2017; Owens et al., 2015). Having demonstrated incremental validity, the patented BASC-3, BESS and the freely available PSC-17 warrant further investigation to determine whether their multi-informant predictive validity are found to be acceptable. It is important to note that the widely used SDQ did not meet acceptable standards for predictive validity and as such, requires further research prior to being used for UMHS purposes in preschool children.

Based on the limited research examining incremental validity of preschool UMHS measures, it would appear that educator ratings are important and add significant incremental value to identifying children where problem behaviour occurs across multiple settings. Given how resource-intensive the implementation of multi-informant UMHS is, there is a strong need for further evidence that examines the incremental validity and effectiveness of UMHS. Future studies that investigate incremental validity need to carefully select gold-standard comparison measures which span various child contexts so that one informant is not inadvertently favoured over others (Haynes & Lench, 2003).

An important implication for future research is the examination of measures in terms of their ease of implementation and use in clinical practice. Some research in the area of measurement-based care has differentiated between the constructs of clinical utility and validity, defining “clinical utility” in terms of ease of implementation and usefulness for clinicians, and “validity” in terms of the evidence for specific measures (McLeod et al., 2022). If adopted in UMHS, this distinction may be of value as the ease of implementation and usefulness for clinicians and non-clinicians such as educators may be an important factor as to whether UMHS is more widely implemented.

Moreover, there is a need for additional studies examining UMHS, especially those which are representative and broader in scope. Large, population-level studies can provide important normative data that are inclusive of different cultural groups, children in urban and regional contexts and validated across ages. Thus, the results of this review suggest there continues to be a need to examine existing measures and develop new multi-informant UMHS measures for preschool-aged children that are psychometrically valid and reliable, accurate for identifying children at risk of MH difficulties. Such research should also enable further examination of acceptability of UMHS in preschool settings.

## Conclusion

In examining the available UMHS measures utilised in preschool-aged children by parents and educators and by evaluating the psychometric evidence presented in studies, this review found promising results in terms of the clinical utility of measures to identify child MH concerns. However, with few measures reporting on all constructs of interest, there remains a need for further research on clinical utility, effectiveness and acceptability of measures. Accurate identification of young children at risk of MH problems is critical to providing early intervention and disrupting the trajectory of MH problems, and research to identify valid screening measures that can be easily implemented by teachers and educators is an important first step.

## Supplementary Information

Below is the link to the electronic supplementary material.

Supplementary file 1 (DOCX 21 kb)
