# Supplementary material for: Multi-Informant Universal Mental Health Screening for Preschool-Aged Children by Parents and Educators: A PRISMA Systematic Review
Source: Clin Child Fam Psychol Rev. 2024 Nov 14;28(1):1–21. doi: 10.1007/s10567-024-00506-2 (PMC11885398; doi:10.1007/s10567-024-00506-2)
Supplement: Supplementary file 1 — (DOCX 21 kb) [file 10567_2024_506_MOESM1_ESM.docx]

# Supplementary file 1.

**Codebook headings**

Year (published)

Country

Study design

Main aim/goal of study

Sample size

Children n =

Educators n =

Parents n =

Gender for children (% of sample)

female

male

non-binary

Age range of children

Mean age of children

Study population

Study setting, number & type

Mental health domain screened

Recruitment criteria: inclusion and/or exclusion

Recruitment strategy/procedure

Demographic characteristics for children

Demographic characteristics for parents

Criterion measure(s) informant, scale, subscales for comparison measures

Main, screening measures and Informants

Main measure(s) purpose

Administration format

Main measure(s) construct of interest

Main measure age range validated with

Main measure number of items - educator report

Main measure number of items - parent report

Main measure response format

Reliability:

Internal consistency - Parent

Internal consistency - Educator

Test-retest - Parent

Test-retest - Educator

Inter-rater reliability, correlation / cross-informant agreement

Validity:

Predictive validity/diagnostic statistics

Educator-report:

Sensitivity

Specificity

PPV

NPV

Parent-report:

Sensitivity

Specificity

PPV

NPV

Any other predictive validity data?

Incremental validity

Convergent validity with other measures

Any other parent & educator similarities, differences, and discrepancies?

Did screening link to intervention or referral?

Effectiveness data about the screening

Acceptability data - Parents

Acceptability data - Educators

# Supplementary file 2.

**Table 4**

*Psychometric Properties of Measures*

| *Author (Year)* | *Sample*  *N (Educator, Parent)* | *Screening measure*  *(no. of items)* | *Convergent validity* *(Educator, Parent)* | *Inter-rater reliability* | *Internal Consistency*  *(Educator, Parent)* | *Test-retest reliability (Parent, Educator)* |
| --- | --- | --- | --- | --- | --- | --- |
| Barbarin et al., (2007) | Study 1: 238, 415   Study 2: NR, NR | ABLE  (Stage 1: 10, Stage 2: 40) | **Educator:**  NR  **Parent:**  ABLE and ORCB: disobedience .12, *p* < .05  Fearfulness .12, *p* < .05  Aggression .17, *p* < .001  Sadness .11, *p* < .05 | Study 1: 76% of the time parents and teachers agreed on which children were or were not designated a case (i.e., when either informant reported a concern and endorsed two or more severity  items related to that concern.)  Study 2: 77% of the time parents and teachers agreed that a child did not have problems. | NR  NR | NR  NR |
| Doove et al., (2019) | 294  329 | VAS (3)  PEDS– Dutch Version  (10) | **Educator** (baseline, follow-up):  Child competence VAS and C-TRF: −0.40, −0.32, *p* <.001  Child competence VAS and SDQ: −0.47, −0.32, *p* <.001  PEDS NR  **Parent** (baseline, follow-up):  Parenting VAS and CBCL: −0.57, −0.51, *p* <.001  Parenting VAS and SDQ: −0.47, −0.45, *p* <.001  Child behaviour VAS and CBCL: −0.56, −0.57, *p* <.001  Child behaviour VAS and SDQ: −0.46, −0.49, *p* <.001  PEDS NR | NR | NR  0.7 | **Educator:**  Child competence VAS: = 0.9, 95% CI = [0.8, 1.0]  PEDS NR  **Parent:**  Parenting VAS = 0.8, 95% CI = [0.6, 0.9]  Child behaviour VAS = 0.9, 95% CI = [0.8, 1.0]    PEDS: 0.8, 95% CI = [0.5, 0.9] |
| Ezpeleta et al., (2013) | **Phase 1**  N/A,  1,341  **Phase 2**  94,  622 | SDQ  (25) | **Educator:**  SDQ and CBCL Int.: .19  Ext.: .26  Total: .17  SDQ and DICA-PPYC  Int.: .17  Ext.:.35  Total: .26  **Parent:**  SDQ and CBCL Int.: .52  Ext.: .62  Total: .58  SDQ and DICA-PPYC  Int.: .41  Ext.:.56  Total: .54 | Partial measurement invariance: [Δχ2(16)=23.7; p=  0.096], 80% (21 of 25) of the factor loading  parameters were equivalent across parents' and  teachers' reports | **Educator**  **Model 1:**  0.67 (prosocial) - 0.93 (hyper-activity)    **Parents**  **Model 2**:  0.81 (Int) - 0.91 (Ext) | NR  NR |
| Feeney-Kettler et al., (2011) | 112  113 | PBSS  (46) | NR | PBSS Phase 2 Total Score: (*r* = .35, n = 104)  Ext. Symptom Scale scores: (*r* = .52, n = 104)  Int. Symptom Scale scores: (*r* = .14, n = 104)  Prosocial Behavior Scale scores: (*r* = .31, n = 104) | **Composite:**  Int: 0.85  (2 – 3 years);  0.87  (4 – 5 years)    Ext: 0.87  (2 – 3 years); 0.90  (4 – 5 years) | **Educator:**  9 – 70 days apart  Int = 0.85  Ext = 0.86    **Parent:**  8 – 65 days apart  Int = 0.86  Ext = 0.81 |
| Feeney-Kettler et al., (2019) | 122  122 | PBSS  (46) | NR | PBSS Total score: (*r* =.26)  Ext. behaviours scores: (*r* =.27 to.37)  Int. behaviours scores: (*r* = −.09 to.10) | **Educator:**  Int: .90 [.87,.92]  Ext .97 [.96,.98]  Prosocial: .96 [.95,.97]  Total: .97 [.96,.98]      **Parent:**  Int: .85 [.81,.88]  Ext: .94 [.92,.95]  Prosocial: .90 [.87,.92]  Total: .94 [.92, .95] | NA  NA |
| Girio-Herrera et al., (2015) | **Study 1:**  56   12    **Study 2:**   568  273 | IRS  (31) | NR | Study 1:  Teacher IRS overall and parent-rated ext. .25; Teacher IRS overall and parent-rated int. .03; Teacher IRS overall and parent-rated adaptive skills -.29  Parent IRS overall and teacher-rated ext. .16; Parent IRS overall and teacher-rated int. .07; Parent IRS overall and teacher-rated adaptive skills -.11  Study 2: Parent-rated IRS scores and teacher-rated BESS scores: all correlations lower than .24. | NR  NR | NR  NR |
| Kettler et al., (2017) | 105  105 | PBSS  (46) | **Educator:**  PBSS and BESS total scores: 0.89;  PBSS and ASEBA total: 0.83, *p* < .05  **Parent:**  PBSS and BESS total: 0.70, *p* < .05  PBSS and ASEBA total: 0.61, *p* < .05 | PBSS Total Score (r = 0.26)  Nomination rubrics (r = 0.37-0.40)  Rating scales (r = 0.05-0.36) | **Educator:**  Int: 0.88; Ext: 0.96; prosocial 0.96; total 0.97    **Parent:**  Int: 0.87  Ext: 0.94  Prosocial: 0.90  Total: 0.94 | NR  NR |
| Moore, Dowdy, Hinton, et al., (2022) | 40  330 | BASC-3 BESS (86) | NR | NR | NR  NR | NR  NR |
| Moore, Dowdy, Fleury, et al., (2022) | 14  535 | BASC-3 BESS (86); PSC-17 (17) | **Educator:**  PSC-17 and BESS-BERI .89, *p* < .001  **Parent:**  PSC-17 and BESS-BERI .68, *p* < .001 | BESS (*r* = .64)  PSC-17 (*r* = .26)  Total scores Cohen’s kappa:  *κ*(BESS) = .08 (95% CI [−.02, .19]), *p* = .046  *κ*(PSC-17) = .09 (95% CI [−.01, .21]), *p* = .022 | **Educator:**  BASC-3  α = .83  PSC-17:   α = .92    **Parent:**  BASC-3  α = .87  PSC-17:  α = .78 | NR  NR |
| Stefan et al., (2017) | NR  180 | ECS   (30) | **Educator:**  ECS/SCS-T and ASEBA scales: -.69- .09  **Parent:**  ECS/SCS-P and ASEBA scales: -.67- -.08 | ECS-P/ECS-T total scores and subscale scores: .19–.40  SCS-P/SCS-T total scores and subscale scores: .20–.27, | **Educator:**  Emotional competence scale from the ECS-T, α = .84–.90;    SCS-T: α = .92–.94;    **Parent:**  ECS-P α = .74–.80 (emotional competence subscales)    SCS-P: social competence scale from the SCS-P, α = .81–.84, | **Educator:**  ECS-T: .82–.88  SCS-T: .80–.87  **Parent:**  ECS-P: .80–.85  SCS-P: .81–.86. |
| Takayanagi et al., (2016) | NR  838 | ADHD-Rating Scale-IV Parent (18) and Educator versions (18) | **Educator:**  NR  **Parent:**  ADHD-RS total score – SDQ total difficulties: r = 0.70 (p < 0.001) | ADHD-RS total scores: r = 0.27 (p < 0.001) | **Educator:**  Alpha = 0.93 (inattention)  Alpha = 0.93 (hyperactivity-impulsivity    **Parent:**  Alpha = 0.86 (inattention)  Alpha = 0.85 (hyperactivity-impulsivity) | NR  NR |

*Note.* ABLE = Attention Behaviour Language Emotions; ADHD = Attention-Deficit/Hyperactivity Disorder; ASEBA = Achenbach System for Empirically-Based Assessment; BASC-2/3 = Behaviour Assessment System for Children Second/Third edition; BERI = Behavioural and Emotional Risk Index; BESS = Behavioral and Emotional Screening System; CBCL = Child Behavior Checklist; C-TRF = Caregiver-Teacher Rating Form; DICA-PPYC = Diagnostic Interview for Children and Adolescents for Parents of Preschool And Young Children; ECS-P/T= Emotion Competence Screening Parent/Teacher Form; Ext = externalising; K = Kindergarten; Int = internalising; IRS = Impairment Rating Scale; NR = not reported; ORCB = Observer ratings of Child Behaviour; PBSS = Preschool Behavior Screening System; PEDS = Parents' Evaluation of Developmental Status; PSC-17 = Pediatric Symptom Checklist-17; SCS-P/T = Social Competence Screening Parent/Teacher Form; SDQ = Strengths and Difficulties Questionnaire; TCRS = Teacher Child Rating Scale; VAS = Visual Analogue Scales.
